# A Phase I Study of the Non-Receptor Kinase Inhibitor Bosutinib in Combination with Pemetrexed in Patients with Selected Metastatic Solid Tumors

**DOI:** 10.3390/curroncol29120744

**Published:** 2022-12-03

**Authors:** Nagla Abdel Karim, Asad Ullah, Hongkun Wang, Mahran Shoukier, Steven Pulliam, Ahmed Khaled, Nikhil Patel, John C. Morris

**Affiliations:** 1 Department of Medicine, Inova Schar Cancer Institute, University of Virginia, Fairfax, VA 22031, USA; 2 Department of Pathology, Vanderbilt University Medical Center, Nashville, TN 37232, USA; 3 Department of Statistics, Georgetown University, Washington, DC 20057, USA; 4 Department of Hematology-Oncology, Georgia Cancer Center, Augusta, GA 30906, USA; 5 Department of Surgery, The University of Tennessee at Chattanooga, Chattanooga, TN 37403, USA; 6 Department of Medicine, Hematology-Oncology, University of Cincinnati, Cincinnati, OH 45219, USA; 7 Department of Pathology, Augusta University Medical Center, Augusta, GA 30906, USA

**Keywords:** adenocarcinoma, chronic myelocytic leukemia, chemotherapy, Bosutinib

## Abstract

Src is overexpressed in various cancers, including 27% of non-small cell lung cancer NSCLC, and is correlated with poor clinical outcomes. We hypothesize that Src kinase inhibitors, including Bosutinib, may exhibit clinical synergy in combination with the antifolate drug pemetrexed. In this Phase I, dose-escalation, safety, and maximum tolerated dose (MTD)-determining study, 14 patients with advanced metastatic solid tumors that had progressed on “standard of care” chemotherapy were enrolled in a 3 + 3 dose escalation study. Oral Bosutinib was administered once daily beginning on day 1, where the first cohort started at an oral dose of 200 mg daily with pemetrexed 500 mg/m^2^ IV on a three-week schedule. The study’s primary objective was to determine the dose-limiting toxicity (DLT), the MTD of Bosutinib in combination with pemetrexed, and the type and frequency of adverse events associated with this treatment. Twelve patients were evaluable for response, including ten patients with adenocarcinoma of the lung, one patient with metastatic adenocarcinoma of the appendix, and one patient with urothelial carcinoma. The median number of Bosutinib and pemetrexed cycles received was 4 (range, 1–4). The MTD of oral Bosutinib in this combination was 300 mg daily. Two patients (17%) had a partial response (PR), and seven patients (58%) showed stable disease (SD) as the best response after the fourth cycle (end of treatment). One patient had disease progression after the second cycle, while three patients had disease progression after the fourth cycle. The two responders and the two patients with the longest stable disease duration or stabilization of disease following progression on multiple systemic therapies demonstrated Src overexpression on immunohistochemical staining of their tumor. The median progression-free survival (PFS) was 6.89 months (95% CI (3.48, 30.85)), and the median overall survival (OS) was 11.7 months (95% CI (3.87, 30.85)). Despite the limitations of this Phase I study, there appears to be potential efficacy of this combination in previously treated patients.

## 1. Introduction

The viral (*v*−) and cellular (*c−) src* (sarcoma) oncogenes are members of a class of oncogenic non-receptor tyrosine kinases originally described by virologist Francis Peyton Rous as a transforming animal virus for which he received the 1917 Nobel Prize [1]. Its mechanism was later elucidated by J. Michael Bishop and Harold Varmus, for whom they were awarded the 1989 Nobel Prize [2]. This class includes the Src, Yes, Fyn, Fgr, Yrk, Lyn, Blk, Hck, and Lck kinases [3,4]. The activation of the Src pathway has been observed in colon, liver, lung, breast, and pancreas cancers. Src is overexpressed in 60–80% of pulmonary adenocarcinomas, bronchioloalveolar cancers, and 50% of squamous cell lung carcinomas [5]. Src is overexpressed in 27% of non-small cell lung cancers (NSCLC0 (*N* = 311) and is correlated with poorer disease-free (*p* = 0.0015) and overall survival (*p* = 0.0008) [6]. The activation of c-*Src* promotes epithelial–mesenchymal transformation (EMT), cell survival, angiogenesis, proliferation and invasion pathways, and tumor growth [6,7]. Common mechanisms of Src kinase activation are a result of mutations that result in their increased activity or their overexpression.

Interestingly, Src activity has been demonstrated to modulate cytotoxicity to drug treatment [8]. In vitro data indicate that Src inhibition decreases resistance to thymidylate synthase (TS)-inhibiting drugs, mainly pemetrexed [8]. Bosutinib (4-[(2,4-dichloro-5-methoxyphenyl)amino]-6-methoxy-7-[3-(4-methylpiperazin-1-yl)propoxy]quinoline-3-carbonitrile; BOSULIF^®^, Pfizer, New York, NY, USA) is an ATP-competitive Bcr-Abl tyrosine-kinase inhibitor (TKI) with inhibitory effects on Src family kinases [9,10]. Bosutinib is a small, orally bioavailable molecule, a dual Src/Abl TKI with minimal inhibitory activity against c-KIT or platelet-derived growth factor receptor (PDGFR). Src inhibitors, mostly dasatinib, have been studied in a number of Phase I/II clinical trials in NSCLC, breast, and prostate cancer [9,10]. Single-agent dasatinib was reported to have modest clinical activity in patients with advanced NSCLC [11]. The combination of cytotoxic chemotherapy and Src signaling pathway inhibitors represents a novel potential strategy to improve therapy. Preclinical data suggest that TS and Src act via a common pathway and that their overexpression has prognostic significance in NSCLC and possibly other tumors [8].

Ceppi et al. explored the concept that Src inhibitors might be synergistic in combination with pemetrexed. Immunohistochemical detection in tumor specimens confirmed that Src kinase activation, evaluated by a phosphospecific antibody, was associated with higher TS expression [8]. Src-inhibiting agents synergistically enhanced pemetrexed cytotoxicity in human A549 lung cancer cells assessed by in vitro MTT and apoptosis assays. The biological explanation for this enhancement was that co-treatment with the Src inhibitor prevented the upregulation of TS messenger RNA and subsequent TS protein levels induced by pemetrexed that increased resistance to pemetrexed treatment [8]. This is highly suggestive that TS and Src likely function in a common pathway and that Src represents a potential target to improve the efficacy of TS-inhibiting agents.

Bosutinib has been used in several clinical settings either as a single agent or in combination with other agents with proven safety [12,13,14]. Bosutinib can be safely administered in combination with a TS inhibitor. Pemetrexed was selectively approved for the treatment of advanced non-squamous NSCLC due to the lack of meaningful response in squamous NSCLC. This was subsequently demonstrated to be on the basis that TS was shown to have low expression in non-squamous NSCLC. Thus, pemetrexed, a TS inhibitor, was more effective in this population than in squamous cell carcinoma of the lung. TS is an enzyme essential for DNA replication, and its overexpression is associated with reduced sensitivity to pemetrexed [15,16].

The protein kinases RAF and SRC are validated therapeutic targets in KRAS-mutant pancreatic ductal adenocarcinomas, colorectal, and non-small-cell lung cancers. Novel therapies such as CCT3833 were shown to inhibit RAF and SRC in KRAS-mutant tumors in vitro and in vivo. It has been evaluated in a Phase I clinical trial (NCT02437227) showing significant prolongation of progression-free survival of a patient with a G12VKRAS spindle cell sarcoma who did not respond to a multikinase inhibitor and therefore had limited treatment options [17].

Despite the reports of a synergistic effect of Src inhibitors with pemetrexed, prospective treatment data on advanced metastatic solid tumors is lacking. Here, we report our experience in patients with advanced metastatic solid tumors who progressed on prior standard-of-care chemotherapy and received Bosutinib in combination with pemetrexed.

## 2. Patients and Methods

### 2.1. Methods

This is a Phase I dose-escalation study, where 14 patients were enrolled with advanced metastatic solid tumors that had progressed on “standard of care” chemotherapy. Twelve out of the fourteen patients were evaluable for clinical outcome after they had received oral Bosutinib in combination with pemetrexed. Bosutinib was administered once daily beginning on day 1 to groups of study patients in a 3 + 3 dose-escalation design. The first cohort started at an oral dose of Bosutinib 200 mg daily for 21 days with IV pemetrexed 500 mg/m^2^ day 1 on a three-week schedule. The primary objective of the study was to determine the dose-limiting toxicity (DLT) and maximum tolerated dose (MTD) of Bosutinib in combination with pemetrexed, and the type and frequency of adverse events associated with this treatment. The secondary objectives were to estimate tumor response rate (RR), progression-free survival (PFS), and overall survival (OS).

### 2.2. Eligibility Criteria

The eligibility criteria were as follows. Patients with previously treated pathologically or cytologically proven advanced metastatic advanced non-squamous NSCLC or malignant pleural mesothelioma (MPM), or those tumors cited in the NCCN Compendium (bladder and urethral cancer, ovarian cancer, primary peritoneal carcinoma, thymoma, and thymic carcinoma, and uterine cervical cancer) for accepted off-label use without a standard curative-intent treatment available to them. Aged 18 years or older. ECOG Performance Status between 0–2, or Karnofsky Performance Score (KPS) > 60%. Have one or more measurable lesions according to the Response Evaluation Criteria in Solid Tumors (RECIST) v1.1. Lesions within a previously irradiated field must have demonstrated progression to be eligible. A life expectancy ≥ 3 months. Normal organ and marrow function as defined by absolute neutrophil count > 1500/mm^3^, platelets > 100,000/mm^3^, total bilirubin < 1.5x upper limit of normal, AST(SGOT)/ALT(SGPT) < 3× upper limit of normal. Serum creatinine within normal institutional limits, OR creatinine clearance ≥ 45 mL/min/1.73 m^2^ for patients with creatinine levels above institutional normal. Women and men of child-bearing potential must agree to use adequate contraception (hormonal or barrier method of birth control or abstinence) prior to study entry and for the duration of study participation, and for 4 months after completion of treatment. Should a woman become pregnant or suspect she is pregnant while participating in this study, she should inform her treating physician immediately, and her participation in the study be immediately terminated. Appropriate measures should be taken to confirm the pregnancy, and arrangements made for prenatal follow-up.

### 2.3. Immunohistochemical Analysis of Patient Samples

Morphologically representative areas from advanced metastatic solid tumors were selected from all included patients. Formalin-fixed paraffin-embedded (FFPE) tissue samples were stained at room temperature with SRC (active) monoclonal antibody Clone 28 (Thermo Fisher Scientific #AHO0051, Waltham, MA, USA) at 1:300 dilution. Counterstaining with hematoxylin was the final step.

### 2.4. Statistical Analysis

Patients’ characteristics and clinical outcomes were summarized using descriptive statistics (Table 1 and Table 2). OS was calculated from the date of starting treatment to the date of death due to any cause. For patients who were alive, their OS was censored at the last follow-up date. PFS was calculated from the date of starting treatment to the date of disease progression, death due to any cause, or last documented follow-up, whichever comes first. The Kaplan–Meier method was used to estimate PFS and OS. SAS software version 9.4 (SAS Ins., Cary, NC, USA) was used for all statistical analyses.

## 3. Results

### 3.1. Demographic Characteristics

Adult patients previously treated for advanced, progressive, or recurrent metastatic solid tumors that are eligible for treatment with pemetrexed were treated in cohorts of escalating doses with Bosutinib and the antifolate, pemetrexed, to determine the maximum tolerated dose of this combination and estimate the antitumor activity of this combination. Both agents are FDA-approved and in current clinical use (Table 1). Twelve of fourteen patients were evaluable for assessment of dosing and response. All study patients had progressed on prior “standard of care “chemotherapy and included ten patients with adenocarcinoma of the lung, one patient with metastatic adenocarcinoma of the appendix, and one patient with urothelial carcinoma. Four patients (33%) had prior pemetrexed exposure. The median age was 63 years (range 58–84). The median number of Bosutinib and pemetrexed cycles received was 4 (range 1–4).

### 3.2. Maximum Tolerated Dose of Bosutinib

Bosutinib was administered in a 3 + 3 Phase I study design as an oral dose of 200 mg once daily for four 21-day cycles within the first cohort without DLT, and then 300 mg in the second cohort without DLT. DLT was defined as a treatment-related CTCAE ≥ Grade 3 non-hematologic toxicity. However, when Bosutinib was administered at 400 mg within the third cohort, two out of three patients experienced DLT of either grade 3–4 elevated AST/ALT that was transient or Grade 3 fatigue. The MTD was determined to be Bosutinib 300 mg daily in combination with pemetrexed 500 mg/m^2^ IV day 1 on a 21-day cycle.

### 3.3. Clinical Outcomes

No treatment-related deaths were seen. For patients who subsequently died, the cause of death was either related to disease progression or other unrelated comorbidities, many of which occurred after the end of the study.

Two patients (17%) had a partial response (PR), and seven patients (58%) had stable disease (SD) as the best response after the fourth cycle (Table 2). One patient had disease progression after the second cycle. A total of three patients experienced disease progression after their fourth cycle. The two responders and the patients with either the longest stable disease duration or stabilization of disease following progression on multiple systemic therapies all demonstrated *Src* overexpression on immunohistochemical staining of their tumor. No deaths related to the treatment were seen. Two patients died of pneumonia and sepsis several months after completion of the study; both demonstrated stable disease while on the study. One patient died after being transferred to a hospice due to disease progression. The remainder of the patients were followed, and subsequent deaths were related to the progression of the disease.

**Figure 1:**
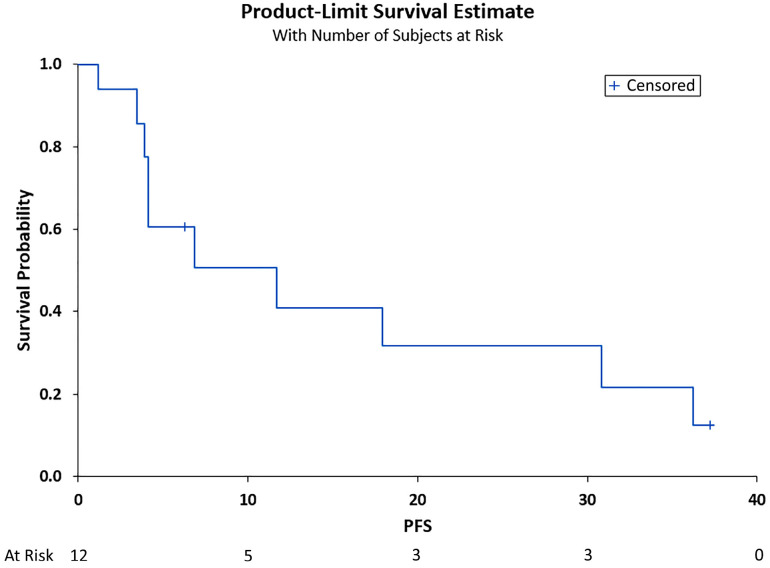
Progression-free survival (PFS) in months. Shown is the PFS after treatment for the total cohort of 12 patients vs. the overall survival probability. Median PFS was 6.89 months (95% CI (3.48, 30.85)).

**Figure 2:**
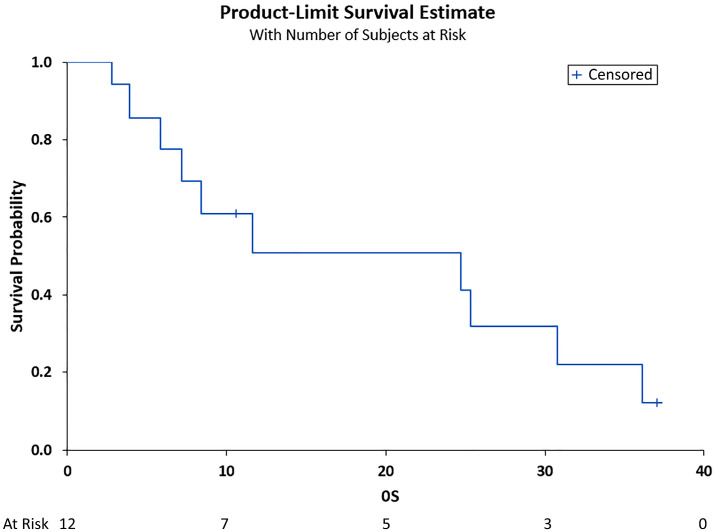
Overall survival (OS) in months. Shown is the OS after treatment for the total cohort of 12 patients vs. the overall survivability probability. Median OS was 11.7 months (95% CI (3.87, 30.85)).

The median PFS was 6.89 months (95% CI (3.48, 30.85)), and the median OS was 11.7 months (95% CI (3.87, 30.85)), as shown in Figure 1 and Figure 2, respectively. Adverse events included pneumonia/sepsis, diarrhea, fatigue, rash, weakness, transaminitis, hypertension, and thrombocytopenia.

#### SRC Expression and Genetic Mutations

Src was found to be overexpressed in four patients (4/12), while the remaining cases did not express Src. Molecular testing for KRAS mutations was caried out on four patients. In one patient, K-RAS G12V was detected; in one case, K-RAS G12C was mutated; in one case, K-RAS was mutant; and one case, K-RAS was negative. All K-RAS-mutated patients had stable disease during the course of the treatment and at the completion of treatment. One patient with a K-RAS mutation-negative tumor had a partial remission. Src overexpressing patients showed variable response rates from partial remission to stable disease (Table 2).

### 3.4. Adverse Events

The most common adverse event noted was fatigue, followed by dyspnea, diarrhea, and rash. Other adverse events included pain in the extremities, constipation, anorexia, peripheral neuropathy, anorgasmia, and dyspepsia. The laboratory events noted were hyperglycemia, hypokalemia, hyperkalemia, hyponatremia, elevated liver transaminases, and neutropenia. These symptoms were mild and without significant morbidity (Table 3).

### 3.5. Laboratory Correlations

**Figure 3:**
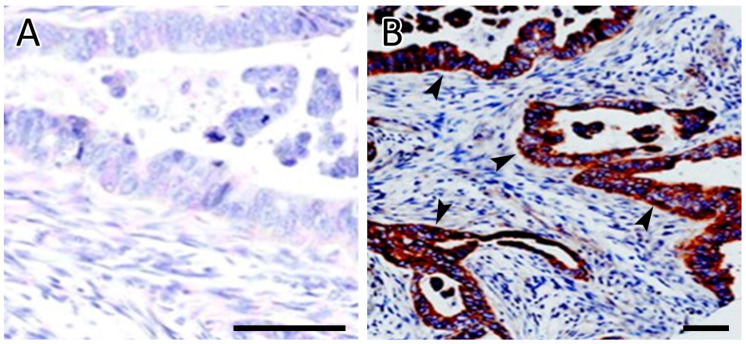
Immunohistochemical analysis of SRC expression. (**A**) Negative staining of tumor cells for Src. (**B**) Strong and diffuse cytoplasmic staining of tumor cells for Src. Arrowheads indicate representative SRC cytoplasmic staining. Scale bars = 50 µm.

Immunohistochemistry (IHC) for Src expression was performed on all cases. Only four cases out of 12 were positive for Src immunostaining. Two cases with positive Src IHC had a partial remission or stable disease and the longest survival. The fourth patient with positive Src IHC had the best response to the treatment and was alive ten months following the start of the study, as shown in Table 2. The remaining eight cases with negative Src IHC expression showed a poor or minor response to the treatment. The negative and positive Src IHC results are shown in Figure 3.

## 4. Discussion

The multitargeted antifolate pemetrexed is approved for the treatment of advanced non-squamous NSCLC due to the lack of meaningful responses in NSCLC of squamous histology. This was subsequently demonstrated to be on the basis that the target of pemetrexed is the TS enzyme. TS was shown to have low expression in non-squamous NSCLC; thus, pemetrexed, a TS inhibitor, was more effective in this population than in squamous cell carcinoma of the lung [15]. TS is an enzyme essential for DNA replication, and its overexpression is associated with reduced sensitivity to pemetrexed [15,16].

Patients with non-squamous NSCLC constitute the majority of patients with NSCLC (65–75%), where pemetrexed-based therapy represents an important first-line and maintenance therapy per NCCN guidelines [18,19]. Pemetrexed also constitutes one of the most important and very few treatment options for malignant mesothelioma. In addition, pemetrexed is considered a reasonable second-line treatment option for metastatic urothelial cancer as well as a subsequent treatment option for metastatic ovarian and uterine cervical cancers (NCCN guidelines).

Bosutinib is currently indicated for the treatment of adult patients with chronic, accelerated, or blast-phase Philadelphia chromosome-positive (Ph1+) chronic myelogenous (myelocytic) leukemia (CML) with resistance or intolerance to prior TKI therapy [20]. The percentage of Src phosphorylation in NSCLC is significant and preclinical data suggest that it may be responsible for the decreased response to pemetrexed; thus, examining Src inhibition by an agent such as Bosutinib may be a reasonable option. The increased expression of Src has been reported in 60–80% of adenocarcinomas and bronchioloalveolar cancers and 50% of squamous cell carcinomas isolated from patients with NSCLC. Pp60c-src has also been found in non-small cell lung carcinomas: in 60–80% of adenocarcinomas and bronchoalveolar cancers and 50% of squamous cell carcinomas [21].

The Src protein kinase is a target for anticancer therapy as it is ubiquitous, exhibiting strong expression in cervical, pancreatic, head and neck, skin, and urothelial tumors and moderate expression in colorectal, stomach, and lung tumors [22]. This protein is also involved in the metastatic processes, as its overexpression facilitates the atypical formation of actin-rich structures on the basal surfaces of cells capable of crossing extracellular barriers. These complexes, in turn, allow the extravasation of cancer cells into the bloodstream and access to secondary sites [23]. Cancer cells are not the only target when it comes to manipulating this protein; however, Src has also been implicated in altering the phenotypes of tumor-adjacent cells. This makes it a potential target for negating tumor-induced compositional changes and associated metastatic processes such as angiogenesis and galvanization of fibroblasts associated with cancer cells [24,25].

On a molecular level, Src is negatively regulated by C-terminal Src kinase (Csk), which phosphorylates tyrosine residues to inhibit the activity of Src. The monitoring of Src expression during tumor progression has revealed that the activity of Src greatly increases, suggesting a modified extraneous control mechanism for phosphorylation that may be important and thus a target for activating Src [26]. The significance of tyrosine phosphorylation as a hindrance to Src kinase activity has been underscored in experiments with knockout mutants that display basal Src activity and oncogenic transformation, a stark contrast from the baseline levels represented in vivo where Src phosphorylation rates approach 90–95% [27].

The link between the invasive nature of cancers and the activation of Src is well established in a multitude of preclinical models, encouraging the investigation of Src inhibitors in clinical trials [28]. This family of proteins represents an appealing target for cancer progression in the advanced stages, particularly given the implication of Src activity in cancer dispersal and infiltration. Src has been shown to control the expression of E-cadherin, an important adhesion molecule involved in the EMT. Studies in Src-inhibited breast cancer cells revealed an increase in E-cadherin with an associated decrease in vimentin, a transition to a prior epithelial phenotype, and a blockade of cancer cell migration [29]. Analogous results were obtained in pancreatic tumor cells where the expression of Src was linked to decreased E-cadherin and elevated vimentin levels [30]. The overexpression of Src is seen in a variety of cancers, with the extent of involvement frequently correlating positively with malignant potential and negatively with patient survival. Depending on the tumor type and location, the expression of Src was found to correspond with cellular differentiation, cancer stage, and, ultimately, metastatic potential.

The overexpression of Src in lung cancers, particularly in non-squamous NSCLC, has significant implications regarding the response of tumors to growth factors and interventions that target these pathways. Src was revealed to play an important role in NSCLC cases when EGFR inhibitors such as erlotinib and gefitinib were shown to suppress its activation. Of note, combination therapy to target both Src and EGFR in NSCLC has been implemented with the Src-EGFR crosstalk inhibitor AC-93253 iodide, which showed promising results by impeding cancer growth and cell motility [31]. Acquired resistance to therapeutics is common. When mutants that resisted EGFR inhibitors arose, experiments performed by Wilson et al. revealed that the Src inhibitor dasatinib was highly effective against these cell lines due to its phosphorylating and inactivating properties as well as its ability to induce apoptosis [32]. Another protein that belongs to the Src family of kinases (SFK) has been shown to have similar implications in cases of NSCLC. When overexpressed, Yes has been shown to induce the expansion of tumor cell lines and increase tumor growth and metastatic potential. Garmendia et al. demonstrated that the in vivo treatment of NSCLC cell lines with dasatinib significantly decreased the tumor burden in cell lines that overexpressed, but had minimal effect on cell lines where the expression of Yes was lower [33]. Thus, protein expression levels may determine which patients will respond to Src inhibitors, and therapy may be targeted accordingly.

The involvement of Src in thyroid cancer has also been explored, with both in vivo and in vitro studies revealing that targeting Src staunches tumor growth and invasion. Due to its prevailing nature in thyroid cancer cells, Src has also been targeted in these cases with dasatinib. Similarly, subjecting lung cancer cells to this drug led to the suppression of metastatic progression and growth. Interestingly, this finding appears to impact those in the advanced stages of disease, as the inhibitory properties of dasatinib relied more heavily on arresting growth in faraway locations instead of suppressing cancer cell distribution [34]. Given that bone metastases are common in the latter stages of thyroid cancer, implementing Src inhibitors in these scenarios could provide another use to an already valuable therapy.

Given its lethal course and propensity for chemotherapeutic resistance, ovarian cancer has been heavily explored from a genetic perspective. The most common subtype, high-grade serous ovarian cancer (HGSOC), has been shown to display the frequent activation of Src, which has been associated with later stages of the disease, chemotherapeutic resistance, and, ultimately, decreased survival [35]. Studies by Kim et al. revealed that silencing Src in ovarian cancer cells in vivo can reduce tumor growth and improve the efficacy of chemotherapy [36]. The expression and activity of Src in different ovarian cancer variants were also assessed by Wiener and colleagues, demonstrating an increased expression of Src in 83% of ovarian cancer cell lines. A concomitant elevation in Src tyrosine kinase activity was also reported in 80% of these genetic over-expressers [37]. Of note, this study reported the low expression and activity of Src in the normal ovarian epithelium, thus underscoring the importance of this mutation in the pathogenesis of ovarian cancer and the need to evaluate patients for this specific mutation when considering targeted therapeutic approaches.

The involvement of Src in urothelial cancers, though not as heavily researched, has been reported in the literature. Levitt and colleagues revealed that Src was responsible for controlling urothelial cancer cell survival in vitro and that by blocking the effects of Src with dasatinib, the viability of these cancer cells was dramatically suppressed [38]. Viktorsson et al. further underscored the role of Src in urothelial cancer cell survival by showing that the inhibition of Src with dasatinib sensitized urothelial cancer cells to treatment with chemotherapeutics [39]. These studies reinforce that Src should be further explored as a targeted therapy and a complementary intervention to increase the effectiveness of chemotherapeutics in the setting of urothelial cancer.

Bosutinib has undergone multiple Phase I/II trials with promising results despite being a novel therapy. A 2012 Phase I study delivered positive results in patients who underwent monotherapy with Bosutinib. Among the study cohort, stable disease was achieved in 22% of patients with pancreatic cancer, 29% of patients with colorectal cancer, and 47% of patients with NSCLC [13]. Given our previous discussion about the involvement of the Src family of kinases in lung cancer, these results underscore the importance of pursuing further studies of Bosutinib, particularly in non-squamous NSCLC. In a Phase II study focused on Bosutinib monotherapy in locally advanced or metastatic breast cancer cases, stable disease was attained in 32.9% of patients, with greater than 20% achieving stable disease beyond 24 weeks. Of note, the subset of patients that were HER2-positive obtained a greater rate of stable disease at 41.7% [40]. The relationship between HER2 and Src has been discussed previously. These results again emphasize the efficacy of Bosutinib in targeting cancers that either overexpress Src or are enhanced by its downstream signaling. Another Phase I study built upon prior monotherapy trials by combining Bosutinib and capecitabine reported encouraging results. Stable disease was achieved in 64% of patients with colorectal cancer and 45% of patients with breast cancer [12].

Other clinical trials using Bosutinib have been terminated due to unfavorable risk–benefit profiles in these combinations. These setbacks have highlighted the need to determine additional biomarkers that better predict the responses to Bosutinib, a task that has been partially achieved in prior trials by displaying positive responses in Src-expressing cancers with Bosutinib, dasatinib, and other small molecule inhibitors. The results from our current study add to this literature and emphasize the efficacy of Bosutinib in NSCLC expressing Src.

A recent review by Ribatti et al. has highlighted an alternative strategy [41] involving the combination of anti-vascular endothelial growth factor (VEGF) with immune checkpoint inhibitors. Tumor cells acquire resistance to anti-angiogenic therapies, ultimately leading to a lack of response and disease recurrence. While alternative pathways to angiogenesis exist, a combination of immune checkpoint inhibitors, such as targeting PD-1, can improve the treatment response. As was noted in the review, the value of targeting this strategy depends on collecting and correctly using panomics data to craft an individualized response to the targeted tumor. While outside the scope of this trial, our IHC data indicate that efficacy can depend on the expression of the appropriate target. An individualized approach considering panomics data is likely to affect efficacy and would be a worthwhile goal of a similar trial.

In addition, not all of the data concerning the details on the K-RAS status or genotypes of all patients to correlate with the clinical outcome or correlation with the SRC overexpression was available. The study was initiated prior to the current FDA-approved K-RAS inhibitor. Given the signal of efficacy and possible predictive marker, it is worth exploring the relation to K-RAS subtypes in relation to SRC and the expansion to a Phase II study.

## 5. Conclusions

The MTD of the oral Src-inhibitor Bosutinib was 300 mg daily in combination with intravenous pemetrexed 500 mg/m^2^ every three weeks. Establishing the combination therapy parameters permits future trials to investigate issues including individualized approaches and other possible agent combinations.

Despite the limitations of this Phase I study, this combination exhibited efficacy when used in previously treated patients with selected cancers. The response rate was 17% in patients with progressive, difficult-to-treat tumors. The disease control rate (PR + SD) was 92% as the best response and 75% at the end of treatment evaluation. The two responders, the patient with the longest duration of stable disease and the one who achieved stable disease following progression on six previous lines of therapies with ten months survival after starting study therapy, demonstrated Src overexpression. The latter patient with Src overexpression had concomitant *K*-RAS pG12V exon 2.

Our study has several limitations, being a Phase I study with a limited number of patients. The weakness of our study is the small sample size of 14 patients. Nevertheless, it achieved its prospectively set endpoint goal of determining the DLT of the combination of the oral Src kinase inhibitor, Bosutinib, with the anti-folate pemetrexed. In addition, the study suggests a possible therapeutic effect and improved survival in previously treated patients.

## Figures and Tables

**Table 1:** Patient demographics data included in the study.

Total # of Patients	12
**Age** Median (Range) Mean (SD)	63 (58, 84) 66.3 (7.99)
**Gender (%)** Male Female	9 (75%) 3 (25%)
**Race (%)** White African American Asian	6 (50%) 5 (41.67%) 1 (8.33%)

Abbreviation: SD—standard deviation.

**Table 2:** Clinical outcome of patients included in the study.

**# of Prior Therapies** Median (Range)	**2 (1–6)**
**# of cycles on study** Median (Range)	4 (2–4)
**Best response (%)** PR SD PD	2 (16.7%) 1 (8.3%) 9 (75%)
**Response at the end of treatment (%)** PR SD PD	2 (16.7%) 3 (25%) 7 (58.3%)
**K-RAS mutation (%)** Negative Mutant G12V G12C NA	2 (16.7%) 1 (8.3%) 1 (8.3%) 1 (8.3%) 7 (58.3%)
**SRC (%)** Positive Negative	4 (33.3%) 8 (66.7%)

Abbreviations: K-RAS—Kirsten rat sarcoma virus; NA—not available; PD—progressive disease; PR—partial response; SD—stable disease; SRC—sarcoma.

**Table 3:** Persistent toxicities or Grade ≥ 3 serious adverse events (all reported events). DLT related to the study drug was reported within the first 28 days.

Event # of Patients	Grade 1	Grade 2	Grade 3	Grade 4
Fatigue	3	1	1 (DLT)	
Rash	2	1		
Hyperglycemia			1 (Related to disease)	
Hyponatremia		2	1 (Related to disease)	
Hypokalemia		2		
Hyperkalemia	1			
Anorexia		1		
Pain	1			
Weakness				
Dyspnea	3		1 (Related to pneumonia)	
Diarrhea	2	1		
Elevated AST				1 (DLT)
Elevated ALT			1 (DLT)	
Rash		1		
Neutropenia/febrile neutropenia			1	
Anemia			1	
Pain in extremity		1		
Constipation	1			
Peripheral sensory neuropathy	1			
Anorgasmia	1			
Dyspepsia		1		
Esophagitis			1	
Nausea	2			

Abbreviations: ALT—alanine aminotransferase; AST—aspartate aminotransferase; DLT—dose-limiting toxicity.

## Data Availability

Data are available on request from the corresponding author.
